# High-resolution profiling of human cytomegalovirus cell-free DNA in human plasma highlights its exceptionally fragmented nature

**DOI:** 10.1038/s41598-020-60655-6

**Published:** 2020-02-28

**Authors:** Vikas Peddu, Benjamin T. Bradley, Amanda M. Casto, Raj Shree, Brice G. Colbert, Hong Xie, Tracy K. Santo, Meei-Li Huang, Edith Y. Cheng, Eric Konnick, Stephen J. Salipante, Keith R. Jerome, Christina M. Lockwood, Alexander L. Greninger

**Affiliations:** 1 Department of Laboratory Medicine, University of Washington, Seattle, WA USA; 2 Department of Medicine, University of Washington, Seattle, WA USA; 3 Department of Obstetrics and Gynecology, University of Washington, Seattle, WA USA; 4 Fred Hutchinson Cancer Research Center, Seattle, WA USA

**Keywords:** Clinical microbiology, Virology

## Abstract

Human cytomegalovirus (CMV) infections comprise a leading cause of newborn impairments worldwide and are pervasive concerns among the immunocompromised. Quantification of CMV viral loads is increasingly used to guide definitions of CMV disease but standardization of CMV quantitation remains problematic, mostly due to differences in qPCR amplicon sizes between clinical laboratories. Here, we used plasma cfDNA sequencing data from 2,208 samples sent for non-invasive prenatal aneuploidy screening to detect CMV and precisely measure the length of CMV fragments in human plasma. CMV reads were identified in 120 (5.4%) samples. Median cfDNA fragment size derived from CMV was significantly shorter than cfDNA derived from human chromosomes (103 vs 172 bp, p < 0.0001), corresponding to the 3^rd^ percentile of human cfDNA. Sequencing of cfDNA from seven plasma samples from transplant patients positive for CMV confirmed the extraordinarily short nature of CMV cfDNA fragment size with a median length of 149 bp. We further show that these high-resolution measurements of CMV DNA fragment size accurately predict measured discrepancies in serum viral load measurements by different qPCR assays. These results highlight the exceptionally fragmented nature of CMV cfDNA and illustrate the promise of plasma cfDNA sequencing for quantitating viral loads through detection of fragments that would be unrecoverable by qPCR.

## Introduction

Human cytomegalovirus (CMV) is the most common cause of congenital defects in the United States and affects roughly 0.5–1.3% of live births worldwide^1,2^, causing more congenital disease than all disorders tested for in newborn screening combined^3^. Congenital CMV is associated with a variety of late-onset permanent disabilities such as hearing loss, microcephaly, vision defects, and intellectual deficits^4^. Currently, prenatal screening for congenital CMV is not routinely recommended in the United States due to difficulties in serological interpretation as well as the low prevalence of high-risk primary maternal CMV infection and high prevalence of recurrent maternal CMV infection which carries a low risk of transmission to the fetus^5–8^. Congenital CMV infection is instead generally detected by testing saliva or urine specimens from newborns via CMV PCR^9^. Recent work has shown maternal CMV DNAemia to be an independent risk factor for the development of fetal CMV infection, suggesting the possibility for earlier detection^10^.

Non-invasive prenatal testing (NIPT) via sequencing of maternal cell-free DNA (cfDNA) has revolutionized the ability to screen for fetal aneuploidies, subchromosomal copy number alterations, and other genetic diseases^11,12^. cfDNA consists of short, free pieces of DNA derived from across the body that freely circulate in plasma with a short half-life^13^. To date, NIPT has generally been limited to high-risk pregnancies, though there is considerable interest in extending it to lower risk populations^14^. The growth of NIPT offers the ability to screen maternal plasma for CMV and other congenital pathogens through a metagenomic approach without any additional testing. CMV has previously been detected in cfDNA sequencing data, including NIPT^15–18^.

cfDNA sequencing data also offers an opportunity to precisely measure the CMV DNA fragment length in plasma, especially since almost all CMV DNA in plasma exists as free DNA^19^. Quantitation of CMV viral loads is critical for monitoring and guiding treatment of CMV disease in the immunocompromised^20^. Despite the development of an international standard, accurate quantitation of CMV in plasma has remained problematic^21^. Variation in CMV viral loads between different clinical laboratories on the same specimen be up to 100-fold, complicating the ability preventing the development of thresholds to drive treatment decisions^22,23^. A major determinant of variation is the size of the PCR amplicon used in the assay design, as assays with amplicon sizes ≤86 bp have higher results than those with amplicons sizes ≥105 bp^22,24^. While this intuitively makes sense – PCR reactions cannot amplify templates shorter than their targeted amplicon size – to date, there exists no precise measurement of the fragment length of CMV DNA in plasma. Prior work to determine CMV fragment size has relied upon select qPCR of template DNA using different amplicon sizes, which is inherently limited in its ability to profile CMV fragment size at high resolution^19,22,25^.

At our institution, we have performed clinical screening for fetal aneuploidies by cfDNA since May 2017. These methods include paired-end sequencing that allows us to measure cfDNA fragment length to accurately determine fetal fraction. Here, we examined cfDNA sequencing data from 2,208 samples for CMV and a related betaherpesvirus, human herpesvirus 6, as well as 7 additional known CMV-positive plasma specimens taken from solid organ transplant patients.

## Methods

### Study population

We included all maternal plasma samples collected between May 2017 to November 2018 for clinically-indicated aneuploidy screening performed at the University of Washington Department of Laboratory Medicine. The 2,208 cfDNA samples in our cohort were derived from pregnant women in the University of Washington (UW) Medicine network. Of these, 727 tests were performed during validation and 1,481 tests were performed during the clinical implementation phase. Metadata was available for the 1,325 patients (1,481 tests) screened during the clinical implementation phase (Supplemental Table 1). A minimum gestational age of 10 weeks was required for testing. Maternal and neonatal clinical histories were gathered for those samples with a CMV cfDNA fragments per million sample reads (FPM) > 0.3 (10 samples, 9 patients) or those with a CMV cfDNA FPM < 0.3 and a positive qPCR result (2 samples, 2 patients). We collected maternal age, gravidity, parity, preexisting comorbidities, and results from ultrasound studies. Neonatal information included gestational age at birth, birth weight, APGAR scores, and mode of delivery. Additional information gathered from the antenatal period included admission to the neonatal intensive care unit (NICU), length of stay, and any infectious disease testing performed. The seven plasma specimens from solid organ transplant patients were chosen based on CMV viral loads >1,000 IU/mL and availability of >2 mL of excess frozen plasma after clinical testing was performed. This study was approved by the University of Washington Institutional Review Board with a waiver for informed consent based on minimal risk (45 CFR 46.116) and all research was conducted in accordance with United States federal regulations.

### Non-invasive cell-free DNA sequencing and PCR confirmation of CMV

cfDNA reads from plasma were generated through a validated, laboratory-developed method used to screen for fetal aneuploidies and copy number alterations. For sample preparation, whole blood from Streck (BCT1) tubes was centrifuged and plasma was isolated as per the package insert. cfDNA was extracted from plasma using the QIAsymphony Circulating DNA Kit. Following measurement of the DNA concentration in the eluate, next-generation sequencing library preparation was performed on the BioMek 4000 using the KAPA HyperPrep kit for adapter and index ligation. The library was purified using the Agencourt AMPureXP kit prior to amplification. Following amplification, the library was purified on the Agilent BRAVO workstation using AMPure beads. Sample pools were created using an equimolar strategy and diluted to 1 nM. Sequencing was performed using an Illumina NextSeq 500 with a 37 bp paired-end read configuration to enable fragment length determination.

### PCR detection of CMV from cell-free DNA

The 10 high positive samples, a random selection of 32 low positives, and 25 run-matched negative controls were analyzed by qPCR to confirm CMV detection. Copy number for positive samples are presented in Supplemental Table 2. Remnant cfDNA from the original maternal plasma extraction was diluted to obtain 50 µL of DNA. 15 µl of sample was loaded into a 96 well plate with 17.5 µl of Bio-Rad Ssoadvanced Universal Probes Supermix. Plates were sealed, mixed, and vortexed prior to amplification on an Applied Biosystems QuantStudio 7 Flex for 46 cycles at 50 C for 2 minutes, 95 C for 23 seconds, and 60 C for 30 seconds. Samples were run using primers and probes specific for a 66-bp amplicon in the gB/UL55 gene and an 84-bp amplicon in the IE EX-4/UL123 gene of CMV and human β –Globin (Supplemental Table 3). Copy number per milliliter was calculated using a standard curve. Cycle thresholds were compared to the FPM values generated from the bioinformatics pipeline using linear regression.

### Cell-free DNA bioinformatics pipeline for detection of CMV and HHV-6

Code for the analysis is available at https://www.github.com/vpeddu/CMV-NIPT/reproducibility. Briefly, paired-end 37 bp cfDNA reads were aligned against the human herpesvirus 5 Merlin strain reference genome (NC_006273.2), hg38, and telomere-trimmed versions of HHV-6A (NC_001664.4) and HHV-6B (AF157706.1) using bowtie2. cfDNA reads from HHV-6A/B-positive samples were also aligned to portions of coding sequence for human genes EDAR (AH008077.2) and beta-globin (AH001475.2). Normalized depth of coverage was calculated by dividing all values by the highest RPKM determined and multiplying by 100.The resulting bams were sorted using samtools sort and deduplicated using Picard MarkDuplicates^26^. The deduplicated alignment file for CMV was filtered to exclude any aligned reads with fewer than 34 exact matches to the CMV reference genome. Low complexity reads were removed using RepeatMasker and reads were confirmed as CMV via BLASTn alignment to NCBI nucleotide database, selecting for any reads with at least 97% identity and a minimum e-value of 1e-5^27,28^. CMV levels by cfDNA sequencing were quantified as CMV-specific FPM, calculated from the deduplicated fragment counts. A threshold of greater than or equal to 0.3 FPM was set as high positive while any value greater than zero and less than 0.3 FPM was set as low positive. Fragment length was calculated from the insert size column of the deduplicated bam file, parsing fragment sizes between 1 and 499 bp. Statistics and graphical plotting were performed in R using ggplot2, ecdf, t.test, and Kolmogorov–Smirnov statistical tests^29^. For median cfDNA fragment size comparison and cumulative frequency distribution graphing, human reads were randomly downsampled to the same number as the CMV reads and statistical tests performed over 10,000 iterations.

### Simulation of CMV viral loads for qPCR amplicons of different length

In order to model the affect of amplicon length on CMV viral load quantitation, we simulated the use of different qPCR amplicon lengths on the fragment length distribution of CMV measured in this study. We performed two separate groups of simulations based on the fragment length distribution seen in our NIPT cohort (individual 121R04) and transplant cohort. For each simulation run, we randomly selected a 50–350 bp segment of the CMV genome in steps of 50 bp to represent the qPCR amplicon size. We then randomly generated a set of 1,000,000 segments of the CMV genome with the length distribution based on cfDNA sequencing. Assuming a perfect qPCR efficiency, the simulation then determined a corresponding viral load by quantifying the number of fragments in the sample that contained the given amplicon. For each simulation, we generated a total of 1,000 model sample sets and 1,000 corresponding viral loads and viral loads were visualized using the ggplot2 R package^29^.

## Results

### Characteristics of study population and testing performance

The median gestational age for the 1,322 patients (1,481 samples) tested during the clinical implementation phase was 13 weeks and 6 days. Average maternal age was 34 years 6 months. The most common indication for testing was advanced maternal age, accounting for 58.3% of cases. Demographic features of patients tested are listed in Supplemental Table 1.

For the 2,208 maternal specimens sequenced in the study, the median number of total reads per sample was 26,563,081 (central 95^th^ percentile of 6,770,026 to 70,439,008). Variation in read depth was related to the number of samples batched per sequencing run, ranging from five to twenty-seven.

### Cytomegalovirus detection in cfDNA

**Figure 1:**
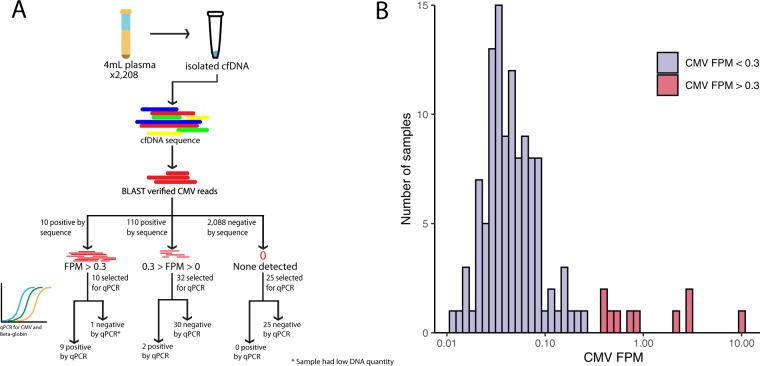
cfDNA pipeline and results. (**A**) Specimen handling and workflow for detecting and confirming CMV cfDNA. (**B**) CMV read distribution by sample. An arbitrary threshold of 0.3 CMV fragments per one million reads (FPM) was set to classify specimens as high (red) or low positive (blue) for subsequent confirmatory qPCR testing.

A total of 120/2208 samples (5.4%) contained at least one read mapping to CMV (Fig. 1A). 110 were subsequently defined as low positives (0 < FPM < 0.3) and 10 were defined as high positives (FPM > 0.3). The distribution of FPM values of the CMV-positive cfDNA samples is shown in Fig. 1B. Verification by qPCR testing was performed on all 10 high positive samples, a subset of low positive (n  =  32), and run-matched negative controls (n  =  25). qPCR detected copies of CMV in 9/10 high positive samples, 2/32 low positives, and 0/25 negative controls. The control samples were appropriately negative for CMV and positive for beta-globin.

Clinical outcomes were only available in five maternal-fetal pairs that tested positive for CMV by qPCR. Fetal outcomes included elective termination for trisomy 21, preterm delivery at 20 weeks gestation, and three live births. Two of the live births were uncomplicated full-term vaginal deliveries, while the other delivered via Cesarean section at 32 weeks gestation due to decreased fetal movement and non-reassuring fetal status. This preterm infant had low APGAR scores and was admitted to the neonatal intensive care unit for prematurity and respiratory distress. The infant was discharged home after 40 days of hospitalization without further apparent complications. In none of the above cases was CMV PCR performed on the mother or neonate.

### The exceptionally short nature of CMV nucleic acid in human plasma cfDNA

Previous work has suggested that microbial cfDNA fragment length may be shorter than that derived from humans^30^. We took advantage of the paired-end reads in our large screen of maternal cfDNA for CMV to estimate the fragment size of CMV cfDNA. In order to attain sufficient reads to estimate the fragment length of CMV cfDNA, we used the cfDNA sample (121R04) with the highest percent CMV measured by sequencing and sequenced it to a total depth of 498 million paired-end reads. Of these, 4,321 paired reads mapped to CMV by bowtie and BLASTn analysis, of which 2,055 fragments remained after deduplication. Reads obtained from our pipeline generally aligned across the length of the CMV genome but had a noticeable lack of coverage in the RL12-RL13-UL1 region, most likely due to sequence diversity in circulating strains of CMV relative to the Merlin reference genome (Supplemental Fig. 1).

**Figure 2:**
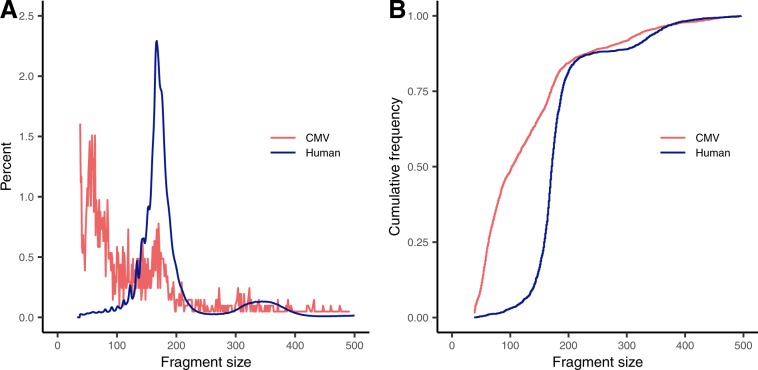
CMV cfDNA fragment size from maternal cfDNA sample 121R04 is significantly shorter than that of human cfDNA. The median fragment length for CMV cfDNA was 103 bp [IQR 63–170 bp], while that of human cfDNA was 172 bp [IQR 158–190 bp], as shown in histogram (**A**) and cumulative distribution plots (**B**).

We found that the median CMV fragment length in cfDNA was significantly shorter than that of human-derived cfDNA (103 v. 172 bp, Wilcoxon test p = 5.4e-178), placing it at the 3^rd^ percentile of human cfDNA fragment size (Fig. 2A). The distribution of CMV cfDNA fragment size [IQR 63–170 bp] was also significantly different than that of human-derived cfDNA [IQR 158–190 bp] (Kolmogorov–Smirnov test, p < 2.2e-16) (Fig. 2B). For the 9 remaining CMV high positive samples, the median fragment length of the 623 combined fragments across all samples was 106 bp [IQR 60–164 bp]. For the 146 CMV DNA fragments taken across all 110 low positive specimens for which both read pairs mapped to CMV, the median fragment length measured 95 bp [IQR 59–161 bp].

### Confirmation of short fragment length of CMV cfDNA in solid organ transplant patients

**Figure 3:**
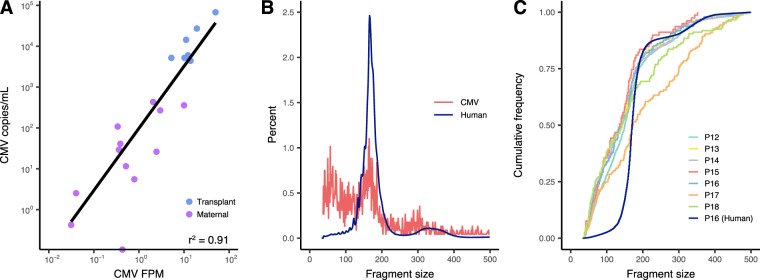
Validation of highly fragmented CMV cfDNA in CMV-positive solid organ transplant patients. Seven CMV-positive specimens from three solid organ transplant patients were subjected to the same cfDNA sequencing protocol as the prenatal plasma samples above. Correlation between CMV qPCR and cfDNA sequencing FPM was high (R^2^ = 0.91) (**A**). CMV DNA fragments from all seven specimens were shorter than that of human cfDNA with an overall median fragment length of 149 bp (**B**). CMV fragment length distribution was similar among the seven specimens, with a flatter size distribution for the sample with the lowest CMV viral load (P17) (**C**).

In order to confirm the short fragment length seen for CMV in plasma for prenatal testing, we sequenced cfDNA from seven plasma specimens taken from three different solid organ transplant patients that tested positive for CMV (Supplemental Table 4). Plasma CMV viral loads ranged between 1,100–17,000 IU/mL (or 4,400–68,000 copies/mL based on a conversion metric of our in-house qPCR to the international standard). When combined with the prenatal CMV testing data, CMV viral loads by qPCR showed an excellent correlation with CMV FPM in cfDNA (r^2^  =  0.91) (Fig. 3A). The size of all CMV-derived fragments in cfDNA from these transplant patients had a median of 149 bp [IQR 86–194 bp], while the human-derived fragments had a median of 170 bp [IQR 158–187 bp] (Fig. 3B). The individual variation in CMV-derived fragment size was low, except for the specimen with the lowest viral load, which had an overall larger size with a median fragment length of 173 bp (Fig. 3C). The CMV reads from transplant patients were also scattered across the CMV genome, again with a lack of coverage in the RL12-RL13-UL1 region (Supplemental Fig. 1).

**Figure 4:**
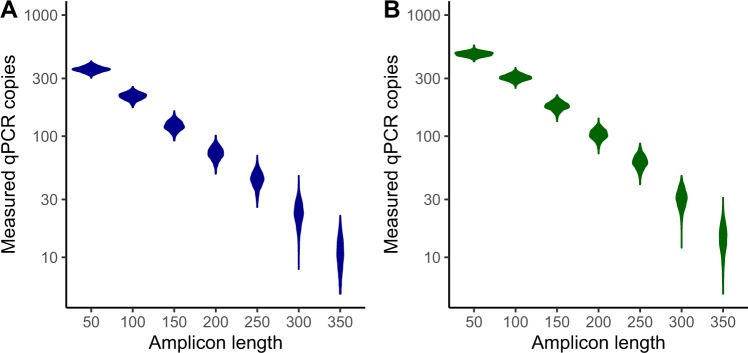
Results of simulation modeling CMV viral loads as determined by qPCR assays with amplicons of different length. For each amplicon length, 1,000 estimated viral loads are displayed corresponding to 1,000 simulated CMV sample sets with fragment length distribution as observed for sample 121R04 (**A**). The same simulation was performed based on a CMV DNA fragment length distribution seen in the combined sample of seven specimens taken from three solid organ transplant patients (**B**).

Previous work using select qPCRs with amplicons between 52–340 bp had shown that amplicon size was a major determinant of variation in CMV viral load testing^22^. Using our high-depth, single-nucleotide resolution measurements of CMV fragment size in plasma cfDNA, we created a simulation that randomly generated target amplicons of various lengths and CMV fragment sets containing 1,000,000 total fragments with fragment length distribution corresponding to (1) that observed for the single sample,121R04, and (2) that observed for the combined sample of seven plasma specimens taken from three solid organ transplant patients. Our simulations show the measured plasma viral load is strongly affected by the qPCR amplicon length based on the distribution of fragment lengths we recovered in both sets of samples, recapitulating the results from prior resource-intensive, multi-center work^22^ (Fig. 4A,B).

### HHV-6 detection in cfDNA

**Figure 5:**
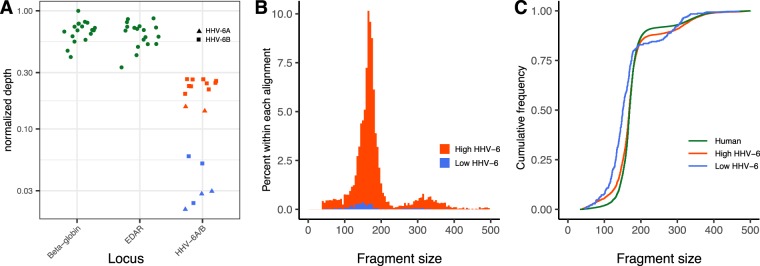
Eighteen samples had HHV-6 cfDNA present, of which twelve had higher levels consistent with iciHHV-6 and six had lower levels relative to human housekeeping genes, beta-globin and EDAR (**A**). The HHV-6 species with the most number of reads for each specimen is depicted by triangles (HHV-6A) or squares (HHV-6B). The fragment length distribution of the HHV-6 cfDNA from the high cluster mirrors that of human cfDNA (**B**). The median fragment length of cfDNA for the high HHV-6 cluster cfDNA was 168 bp [IQR 150–183 bp], while that of low HHV-6.

In order to compare cfDNA detection, quantitation, and fragment length in the maternal plasma to another betaherpesvirus to control for CMV-specific biology, we also looked for HHV-6 in our NIPT cfDNA sequencing data. 18 maternal cfDNA samples from 17 different individuals had reads aligning to HHV-6A or HHV-6B and were classified as HHV-6 positive. Of these 18 positive samples, 12 had a significantly higher ratio of genomic copies of HHV-6:human genome copies (EDAR or beta-globin), likely consistent with inherited chromosomally-integrated HHV-6 (Fig. 5A)^31^. The median fragment length of HHV-6 cfDNA by NIPT sequencing across all positive samples was 167 bp [IQR 147–183 bp], approximating that of human-derived cfDNA. When we specifically compared the fragment length of the twelve high and six low HHV-6 samples, the six low level HHV-6 samples had a shorter fragment length (median 146 bp [IQR 104–176 bp]) than the twelve high level HHV-6 samples (median 168 bp [IQR 150–183 bp]) (Fig. 5B,C). These results are most consistent with a model of normal chromatinization of maternal iciHHV-6 DNA in the high level HHV-6 samples, with the shorter fragments of low level HHV-6 deriving from the placenta due to paternal transmission of iciHHV-6 to the fetus.

## Discussion

Here, we use NIPT cfDNA sequencing data from 2,208 samples from a tertiary academic center to interrogate CMV detection and quantitation in NIPT, as well as fragment length in cfDNA. We show that approximately 5% of samples sent for NIPT had detectable CMV-specific reads, which routinely outstripped the analytical sensitivity of our CMV qPCR assay when performed on cfDNA. We also discovered that CMV cfDNA exists in circulation at a smaller fragment size than human chromosomally-derived cfDNA. We confirmed the highly fragmented nature of CMV in cfDNA in seven CMV-positive specimens from solid organ transplant patients. Given the difficulties associated with screening for congenital infections with existing approaches, NIPT for aneuploidies and copy number changes remains a potentially attractive option to simultaneously test for pathogen sequence, especially given that maternal CMV DNAemia is a risk factor for fetal CMV infection^10^. The rapid growth of NIPT also allows for determination of test characteristics of cfDNA sequencing for pathogen detection, albeit in a specific patient population.

By directly measuring the exceptionally fragmented nature of CMV DNA in plasma, we have confirmed a major mechanism contributing to the variation in CMV levels obtained from different qPCR assays in plasma. Despite a recently implemented international standard, variation in plasma CMV DNA levels measured by qPCR across different clinical laboratories has remained high, with variation of up to 100-fold in copy number between assays^32^. Amplicon size has been identified as a major contributor to interassay variability, with larger amplicons having a relatively lower IU/ml as compared to smaller amplicons^22^. Given that amplicons in CMV qPCR assays range in size from approximately 50 to 350 bp, it is reasonable to assume that PCR assays developed for smaller amplicons would more readily amplify CMV cfDNA fragments. As a test of this assumption, we modeled CMV samples with fragment length distributions consistent with our observed results and demonstrated that viral loads would indeed be expected to decrease with increasing amplicon length. While our model is based on observations from a limited number of samples, previous studies have indicated that the near entirety of CMV DNA in plasma exists as fragmented, virion-free DNA^19,25^. We also note that even the shortest amplicon length trialed in this simulation could not detect the smallest fragments of CMV recovered by cfDNA sequencing. Our results also help explain why fragmentation of CMV international standards to a mode size of approximately 150 bp results in a significant decrease in variance in CMV quantitation among different clinical laboratories, as was recently reported^33^. Furthermore, our results suggest that sequencing fragments <100 bp may be a simple method to enrich for pathogen sequences and comparatively deplete human sequences in cfDNA^30,34,35^.

It is unclear why CMV fragment size was substantially shorter in the NIPT cohort compared to the transplant group. We hypothesize the difference could be due to the higher viral load seen in transplant patients and greater rate of production of longer CMV fragments or differences in the epigenetic state of CMV in different hosts or states of reactivation. Alternatively, changes in host immune pressure may affect the kinetics of cfDNA production and degradation, as different mechanisms of cell death may affect the length of DNA fragments released into the blood^36^.

We confirmed all but one of the specimens with cfDNA FPM > 0.3 by conventional qPCR testing. This threshold was also seen by Chesnais *et al*. (2018) for cfDNA samples that could be confirmed by CMV qPCR^15^. They also detected 2 CMV positives out of 574 samples (0.35%), consistent with our approximate prevalence of 10 high positives out of 2,208 samples (0.45%), albeit in different patient populations. In our one maternal cfDNA high positive sample that tested negative by qPCR, the beta-globin signal was 1.0 log_10_ lower than the next lowest positive sample and had the lowest amount of residual DNA available after cfDNA sequencing. This finding argues that the negative CMV qPCR result was a function of low input DNA as opposed to a true negative.

Of the low positive maternal specimens (FPM < 0.3), only 2 of the 32 tested were positive by qPCR. Nonetheless, we believe these low positive samples were likely CMV positive. All reads were confirmed as CMV by best hit by blastn against the NCBI Nucleotide database and mapped across the CMV genome. No two samples had the same CMV read between samples, arguing against cross-contamination either within the lab or on the sequencer via index hopping. All DNA extractions, library preparations, and sequencing were performed in our clinical genetics laboratory, where no prior CMV work has been performed. Furthermore, the median fragment length of CMV DNA in low positive samples was consistent with the short fragment length observed in CMV positive samples. We note our in-house qPCR uses relatively short amplicon lengths of 66 and 84 bp, which should reduce affects of fragment length on analytical sensitivity. The potential relative insensitivity of qPCR may be related to an insufficient number of DNA fragments in the sample spanning the full length of the amplicon. Alternatively, these false negative qPCR results may be due to the low volumes of DNA available after cfDNA sequencing.

To demonstrate that our approach has the robustness to identify other viral pathogens, we also interrogated the cfDNA sequencing data for evidence of HHV-6. We identified 18 HHV-6 positive samples, 12 of which appeared consistent with maternal iciHHV-6 based on fragment length and copy number. In the six HHV-6 positive samples with an RPKM value below what is expected in iciHHV-6, the cfDNA size distribution of the HHV-6 reads closely matched that seen in placental cfDNA^37^. We hypothesize the shorter read distribution arising in this population is the result of iciHHV-6 from placental DNA fragments. More work will be needed to determine the fragment size of HHV-6 in plasma in the context of primary infection or reactivation.

Our study is significantly limited by the lack of clinical follow-up data. Our hospital system is separate from the major pediatric hospital in our area, complicating our ability to determine postnatal outcomes for any high positive cases subsequently followed locally. While at least one case resulted in prolonged hospitalization due largely to prematurity, no CMV testing was performed and imaging studies did not suggest congenital CMV infection. These factors speak to the difficulty of evaluating NIPT for congenital CMV screening in the context of existing medical care. Our estimation of viral fragment length in cfDNA in NIPT is limited by reliance on one deeply sequenced sample, though we note CMV reads from other NIPT samples showed a similarly short fragment length. Finally, our estimates of CMV fragment length are also currently limited by short-read sequencing approaches, such that we have only estimated fragment lengths shorter than 500 bp and cannot capture potential full-length CMV genomic DNA fragments.

In summary, we demonstrate the presence of CMV in approximately 5% of NIPT cfDNA samples and use this data to show the significantly shorter fragment length of CMV DNA in plasma cfDNA as compared to that of human origin. Despite the potential of NIPT as a diagnostic tool in early detection of congenital CMV, future studies will need to address whether a quantitative level of CMV cfDNA or fragment size distribution predictive of congenital infection exists. We expect the continued growth of cfDNA sequencing and NIPT will enable retrospective evaluation of NIPT data for postnatally confirmed congenital CMV cases in the near future.

## Supplementary information

Supplementary Data.

## References

[1] Kenneson A, Cannon MJ (2007). Review and meta-analysis of the epidemiology of congenital cytomegalovirus (CMV) infection. Rev. Med. Virol..

[2] Dollard SC, Grosse SD, Ross DS (2007). New estimates of the prevalence of neurological and sensory sequelae and mortality associated with congenital cytomegalovirus infection. Rev. Med. Virol..

[3] Ross SA, Boppana SB (2005). Congenital cytomegalovirus infection: outcome and diagnosis. Semin. Pediatr. Infect. Dis..

[4] Hughes BL, Gyamfi-Bannerman C, Society for Maternal-Fetal Medicine (SMFM) (2016). Diagnosis and antenatal management of congenital cytomegalovirus infection. Am. J. Obstet. Gynecol..

[5] Nigro G, Adler SP, La Torre R, Best AM (2005). Congenital Cytomegalovirus Collaborating Group. Passive immunization during pregnancy for congenital cytomegalovirus infection. N. Engl. J. Med..

[6] Lazzarotto T, Guerra B, Lanari M, Gabrielli L, Landini MP (2008). New advances in the diagnosis of congenital cytomegalovirus infection. J. Clin. Virol..

[7] Adler SP (2012). Editorial commentary: Primary maternal cytomegalovirus infection during pregnancy: do we have a treatment option?. Clin. Infect. Dis..

[8] Lazzarotto T, Guerra B, Gabrielli L, Lanari M, Landini MP (2011). Update on the prevention, diagnosis and management of cytomegalovirus infection during pregnancy. Clin. Microbiol. Infect..

[9] Ross SA, Novak Z, Pati S, Boppana SB (2011). Diagnosis of Cytomegalovirus Infections. Infect. Disord. Drug. Targets..

[10] Nigro G. & Adler S.P., Congenital Cytomegalic Disease Collaborating Group. High-dose CMV hyperimmune globulin (HIG) and maternal CMV DNAemia independently predict infant outcome in pregnant women with a primary cytomegalovirus (CMV) infection. *Clin Infect Dis*. (2019). 10.1093/cid/ciz1030 31628849

[11] Lo YM (1997). Presence of fetal DNA in maternal plasma and serum. Lancet..

[12] Lo YM (1998). Quantitative analysis of fetal DNA in maternal plasma and serum: implications for noninvasive prenatal diagnosis. Am. J. Hum. Genet..

[13] Khier S, Lohan L (2018). Kinetics of circulating cell-free DNA for biomedical applications: critical appraisal of the literature. Future Sci. OA..

[14] Gregg AR (2016). Noninvasive prenatal screening for fetal aneuploidy, 2016 update: a position statement of the American College of Medical Genetics and Genomics. Genet. Med..

[15] Chesnais V (2018). Using massively parallel shotgun sequencing of maternal plasmatic cell-free DNA for cytomegalovirus DNA detection during pregnancy: a proof of concept study. Sci. Rep..

[16] Fung M, *et al*. Plasma Cell–Free DNA Next-Generation Sequencing to Diagnose and Monitor Infections in Allogeneic Hematopoietic Stem Cell Transplant Patients. Open Forum Infect Dis [Internet]. [cited 2019 Oct 2]; 5(12). Available from, https://www.ncbi.nlm.nih.gov/pmc/articles/PMC6297859/ (2018) 10.1093/ofid/ofy301 PMC6297859 30581881

[17] Hong DK, *et al*TAMSCN. Liquid biopsy for infectious diseases: sequencing of cell-free plasma to detect pathogen DNA in patients with invasive fungal disease. Diagn Microbiol Infect Dis. (2018). 10.1016/j.diagmicrobio.2018.06.009 30017314

[18] Blauwkamp TA (2019). Analytical and clinical validation of a microbial cell-free DNA sequencing test for infectious disease. Nat. Microbiol..

[19] Tong Y, Pang XL, Mabilangan C, Preiksaitis JK (2017). Determination of the Biological Form of Human Cytomegalovirus DNA in the Plasma of Solid-Organ Transplant Recipients. J. Infect. Dis..

[20] Kotton CN (2018). The Third International Consensus Guidelines on the Management of Cytomegalovirus in Solid-organ Transplantation. Transplantation..

[21] Hayden RT (2017). Progress in Quantitative Viral Load Testing: Variability and Impact of the WHO Quantitative International Standards. J. Clin. Microbiol..

[22] Preiksaitis JK (2016). Are We There Yet? Impact of the First International Standard for Cytomegalovirus DNA on the Harmonization of Results Reported on Plasma Samples. Clin. Infect. Dis..

[23] Razonable RR, Humar A. Cytomegalovirus in solid organ transplant recipients-Guidelines of the American Society of Transplantation Infectious Diseases Community of Practice. Clin. Transplant.; e13512 (2019). 10.1111/ctr.13512 30817026

[24] Valsamakis A (2016). Editorial Commentary: Standardization of Viral Load Testing for Cytomegalovirus: The Long Road Just Got Longer. Clin. Infect. Dis..

[25] Boom R (2002). Human Cytomegalovirus DNA in Plasma and Serum Specimens of Renal Transplant Recipients Is Highly Fragmented. J. Clin. Microbiol..

[26] Langmead B, Salzberg SL (2012). Fast gapped-read alignment with Bowtie 2. Nat. Methods..

[27] Chen N. Using RepeatMasker to identify repetitive elements in genomic sequences. Curr Protoc Bioinformatics.; Chapter 4:Unit 4.10 (2004). 10.1002/0471250953.bi0410s05 18428725

[28] Altschul SF, Gish W, Miller W, Myers EW, Lipman DJ (1990). Basic local alignment search tool. J. Mol. Biol..

[29] Wickham H. Ggplot2: Elegant Graphics for Data Analysis. 2nd ed. Springer Publishing Company, Incorporated; (2009).

[30] Burnham P, *et al*. Single-stranded DNA library preparation uncovers the origin and diversity of ultrashort cell-free DNA in plasma. Sci Rep [Internet]. [cited 2019 Aug 28]; 6. Available from, https://www.ncbi.nlm.nih.gov/pmc/articles/PMC4906518/ (2016) 10.1038/srep27859 PMC4906518 27297799

[31] Sedlak RH (2016). Detection of Human Herpesvirus 6B (HHV-6B) Reactivation in Hematopoietic Cell Transplant Recipients with Inherited Chromosomally Integrated HHV-6A by Droplet Digital PCR. J. Clin. Microbiol..

[32] Hayden RT (2015). Commutability of the First World Health Organization International Standard for Human Cytomegalovirus. J. Clin. Microbiol..

[33] Hayden RT, *et al*. Impact of Fragmentation on Commutability of Epstein-Barr Virus and Cytomegalovirus Quantitative Standards. J Clin Microbiol. (2019). 10.1128/JCM.00888-19 PMC6935903 31619529

[34] Lam WKJ (2018). Sequencing-based counting and size profiling of plasma Epstein–Barr virus DNA enhance population screening of nasopharyngeal carcinoma. PNAS..

[35] Underhill HR (2016). Fragment Length of Circulating Tumor DNA. PLOS Genetics..

[36] Kustanovich A, Schwartz R, Peretz T, Grinshpun A (2019). Life and death of circulating cell-free DNA. Cancer Biol. Therapy..

[37] Sun K (2018). Size-tagged preferred ends in maternal plasma DNA shed light on the production mechanism and show utility in noninvasive prenatal testing. Proc. Natl Acad. Sci. USA.

