# Supplementary material for: High-resolution profiling of human cytomegalovirus cell-free DNA in human plasma highlights its exceptionally fragmented nature
Source: Sci Rep. 2020 Feb 28;10:3734. doi: 10.1038/s41598-020-60655-6 (PMC7048871; doi:10.1038/s41598-020-60655-6)
Supplement: Supplementary file 1 — Supplementary Data. [file 41598_2020_60655_MOESM1_ESM.docx]

**Supplemental Tables and Figure**

**Supplemental Table 1** – Patient demographics for the clinical implementation phase of our cfDNA prenatal screen. Data was collected at the same time as the specimen. A total of 1,481 samples were tested for 1,325 patients. These data do not include patient demographics for samples tested during the validation phase.

|  | Patients Tested (n=1,325) |
| --- | --- |
|  | average |
| **Patient characteristics** |  |
| maternal age (years) | 34.5 |
| gestational age (days) | 98 |
| Height (inches) | 64.3 |
| Weight (pounds) | 162 |
| BMI (kg/m^2^) | 28.1 |
|  |  |
| **Indications for testing** | Number of cases |
| advanced maternal age | 773 |
| abnormal ultrasound | 141 |
| abnormal serum screen | 73 |
| history of increased risk | 33 |
| No indication listed | 66 |
| Other | 239 |
| **Aneuploidies detected** | 35 |
| Common autosome aneuploidies (trisomy 13, 18, 21) | 10 |
| Rare autosome aneuploidies | 9 |
| Subchromosomal deletion | 5 |
| Sex chromosome aneuploidies (monosomy X, XXY, XXX) | 11 |

**Supplemental Table 2** – Quantities for CMV copies per mL plasma for positive prenatal samples identified by cfDNA and analyzed by qPCR. Specimens marked with (*) were identified as low positive samples based on copy number in cfDNA sequencing, while specimen 34P02 (^#^) had fewer than 5µL of DNA remaining.

| **Sample Name** | **CMV**  **copies / ml** | **Beta-globin**  **copies / mL** |
| --- | --- | --- |
| **10P13** | 269.3 | 2096.6 |
| **111P08** | 11.6 | 1024.3 |
| **121R04** | 358.6 | 1324.3 |
| **34P02^#^** | 0.0 | 699.6 |
| **3P13** | 433.8 | 4084.9 |
| **80P04** | 108.4 | 8564.4 |
| **84P05** | 41.0 | 3840.8 |
| **92P02** | 26.2 | 1422.4 |
| **92R11** | 5.6 | 1878.9 |
| **104R** | 29.3 | 495.9 |
| **44P05*** | 2.5 | 1017.5 |
| **96R12*** | 0.4 | 1822.9 |

| Primer | Sequence |
| --- | --- |
| CMVgB primer F | TGG GCG AGG ACA ACG AA |
| CMVgB primer R | TGA GGC TGG GAA GCT GAC AT |
| CMVgB probe | FAM-TGG GCA ACC ACC GCA CTG AGG-TAMRA |
| CMV IE EX-4 primer F | TCC CGC TTA TCC TCR GGT ACA |
| CMV IE EX-4 primer R | TGA GCC TTT CGA GGA SAT GAA |
| CMV IE EX-4 probe | FAM-TCT CAT ACA TGC TCT GCA TAG TTA GCC CAA TAC A-TAMRA |

**Supplemental Table 3** – CMV qPCR primer and probe sequences.

**Supplemental Table 4 –** Sample data for CMV positive plasma specimens from solid organ transplant patients. Our lab has an internal correction for CMV quantities based on IU/mL and our measured copies/mL.

| Sample | Patient | day | solid organ transplant | CMV quantity (IU/mL) | CMV (copies/mL) | CMV  fragments | Total fragments | FPM |
| --- | --- | --- | --- | --- | --- | --- | --- | --- |
| P12 | pt1 | 0 | heart | 17000 | 68000 | 1225 | 24501332 | 50.00 |
| P13 | pt2 | 0 | kidney | 1300 | 5200 | 184 | 17973440 | 10.20 |
| P14 | pt1 | 6 | heart | 6800 | 27200 | 328 | 17023864 | 19.38 |
| P15 | pt1 | 8 | heart | 1300 | 5200 | 81 | 15473729 | 5.28 |
| P16 | pt3 | 0 | liver | 1500 | 6000 | 199 | 16277725 | 12.29 |
| P17 | pt2 | 15 | kidney | 1100 | 4400 | 194 | 13873834 | 14.00 |
| P18 | pt3 | 14 | liver | 3600 | 14400 | 156 | 13992482 | 11.28 |

**Supplemental Figure 1** – Read coverage of the CMV genome from sample 121R04 (a) and from all CMV-positive transplant patients (b). Reads were mapped to the human herpesvirus 5 Merlin strain reference genome (NC_006273.2).

**
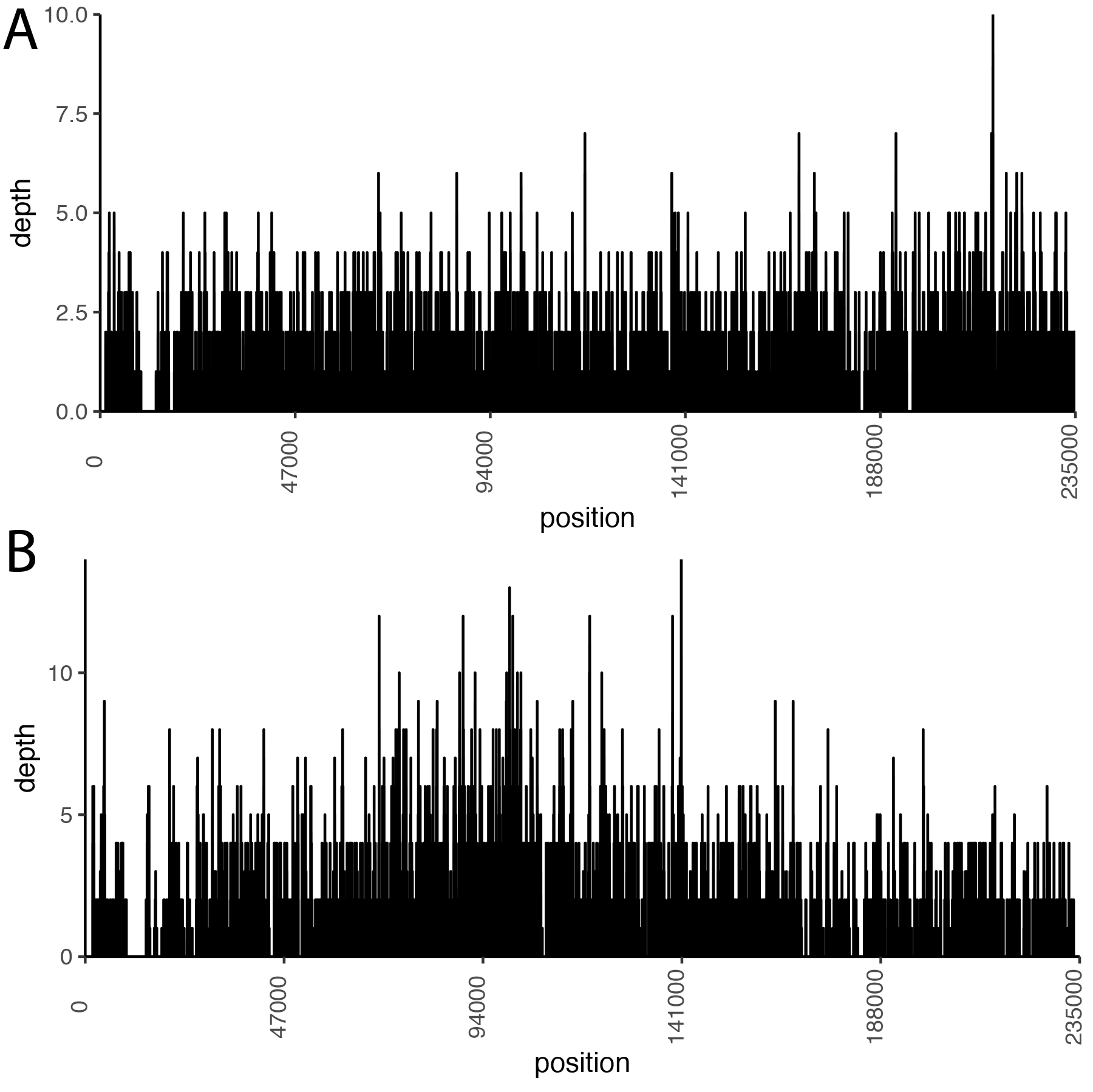
**
